# *Syzygium cumini* Leaf Extract Reverts Hypertriglyceridemia via Downregulation of the Hepatic XBP-1s/PDI/MTP Axis in Monosodium L-Glutamate-Induced Obese Rats

**DOI:** 10.1155/2019/9417498

**Published:** 2019-03-21

**Authors:** Lucas Martins França, Caio Fernando Ferreira Coêlho, Larissa Nara Costa Freitas, Ivana Letícia Santos Souza, Vinicyus Teles Chagas, Victor Debbas, Thais Martins de Lima, Heraldo Possolo de Souza, Francisco Rafael Martins Laurindo, Antonio Marcus de Andrade Paes

**Affiliations:** ^1^Laboratory of Experimental Physiology (LeFisio), Department of Physiological Sciences, Federal University of Maranhão, Av. dos Portugueses, 1966–Cidade Universitária Dom Delgado, São Luís, MA 65080-805, Brazil; ^2^Laboratory of Vascular Biology (LBV), Heart Institute, School of Medicine, University of São Paulo, Av. Dr. Enéas de Carvalho Aguiar, 44–Cerqueira César, São Paulo, SP 05403-900, Brazil; ^3^Laboratory of Medical Investigation (LIM-51), Emergency Medicine Department, School of Medicine, University of São Paulo, Av. Dr. Arnaldo, 455-Cerqueira César, São Paulo, SP 01246-903, Brazil

## Abstract

*Syzygium cumini* is used worldwide for the treatment of metabolic syndrome-associated outcomes. Previously, we described the antihypertriglyceridemic effect of the hydroethanolic extract of *S. cumini* leaf (HESc) in monosodium L-glutamate- (MSG-) induced obese rats. This study sought to investigate the molecular mechanisms underlying the antihypertriglyceridemic effect of HESc in MSG-obese rats. Newborn male Wistar rats were injected subcutaneously with MSG (4.0 g/kg/day, obese group) or saline 1.25% (1.0 mL/kg/day, lean group), from 2nd through 10th postnatal day. At 8 weeks old, obese rats started to be orally treated with HESc (0.5 or 1.0 g/kg/day, *n* = 7) or saline 0.9% (1 mL/kg/day, *n* = 7). Lean rats received saline solution (1 mL/kg/day, *n* = 7). Upon 8-week treatment, animals were euthanized for blood and tissue collection. Another set of adult nonobese Wistar rats was used for the assessment of HESc acute effects on Triton WR1339-induced hypertriglyceridemia. HESc reduced weight gain, as well as adipose tissue fat pads, without altering food intake of obese rats. HESc restored fasting serum glucose, triglycerides, total cholesterol, and free fatty acids, as well as insulin sensitivity, to levels similar to lean rats. Additionally, HESc halved the triglyceride content into very low-density lipoprotein particles, as well as healed liver steatosis, in obese rats. Hepatic protein expression of the endoplasmic reticulum chaperone GRP94 was decreased by HESc, which also downregulated the hepatic triglyceride secretion pathway by reducing the splicing of X-box binding protein 1 (XBP-1s), as well as protein disulfide isomerase (PDI) and microsomal triglyceride transfer protein (MTP) translational levels. This action was further corroborated by the acute inhibitory effect of HESc on triglyceride accumulation on Triton WR1339-treated rats. Our data support the downregulation of the XBP-1s/PDI/MTP axis in the liver of MSG-obese rats as a novel feasible mechanism for the antihypertriglyceridemic effect promoted by the polyphenolic phytocomplex present in *S. cumini* leaf.

## 1. Introduction

Nonalcoholic fatty liver disease (NAFLD) is considered the main hepatic manifestation of metabolic syndrome (MetS) [1]. Under MetS, white adipose tissue hypertrophy causes local insulin resistance that, in turn, increases adipocyte lipolytic activity and decreases local free fatty acid (FFA) recycling, raising serum FFA levels. Increased FFA uptake by hepatocytes leads to ectopic fat accumulation and lipotoxicity due to the limited liver capacity to oxidize and/or export excess FFA [2]. Hyperinsulinemia additionally imposes *de novo* lipogenesis oversizing hepatic fat accumulation, an outcome partially compensated by increased triglyceride (TG) secretion via very low-density lipoprotein (VLDL) particles, that ultimately leads to hypertriglyceridemia [3]. Hypertriglyceridemia is an independent risk factor for cardiovascular diseases, which are the leading cause of morbimortality worldwide [4].

Despite the abovementioned evidences, the molecular mechanisms involved in NAFLD and hypertriglyceridemia onset remain incompletely defined. During the last decade, the endoplasmic reticulum (ER) stress has been proposed as a key player by its role in unfolded protein response (UPR) [5]. UPR occurs when the ER becomes overwhelmed and causes luminal misfolded protein accumulation, eliciting the phosphorylation of three ER transmembrane sensing proteins, namely, inositol-requiring enzyme-1*α* (IRE-1*α*), protein kinase RNA-like ER kinase (PERK), and activating transcription factor 6 (ATF6) [5]. IRE-1*α* subsequently splices the X-box binding protein 1 (XBP-1s) mRNA, a transcription factor importantly involved in the reestablishment of ER homeostasis [6] and hepatic lipogenesis regulation [7]. Studies conducted by us [8] and others [9–11] have demonstrated the importance of the IRE1*α*/XBP-1s pathway for hepatic lipid homeostasis, through either lipogenesis modulation or stimulation of TG secretion, a process mediated by upregulation of microsomal triglyceride transfer protein (MTP) and protein disulfide isomerase (PDI) expression in hepatocytes [8]. Henceforth, the XBP-1s/PDI/MTP axis has emerged as a potential therapeutic target for the treatment of lipid metabolism disorders, especially hypertriglyceridemia [12], despite the plethora of other regulatory targets.

Herbal medicines constitute an important source of bioactive and potentially therapeutic molecules enabled to fulfill a multiple-target strategy for MetS-related outcome treatment [13, 14]. These properties are specially related to their antioxidant capacity, although other mechanisms might feasibly be involved [15]. In this context, cardiometabolic potentialities of *Syzygium cumini* (L.) Skeels (syn: *S. jambolanum* D.C., *Eugenia jambolana* Lam.) have been highlighted [16]. *S. cumini* is an Indian native tree from the Myrtaceae family widely cultivated throughout the world and popularly known as jambolão, jambolan, java plum, or black plum [17]. It is traditionally used to treat a variety of illnesses, most of them related to MetS and its comorbidities [16, 18–21]. Moreover, its ethnopharmacological relevance has been recognized by the Brazilian Ministry of Health, which included *S. cumini* species in the National Index of Medicinal Plants of Interest to the Unified Public Health System, acronym RENISUS [22].

In a previous study, we showed that the hydroethanolic extract of *S. cumini* leaf (HESc) improved the metabolic profile of monosodium L-glutamate- (MSG-) induced obese rats, especially by reverting TG accumulation in both the liver and serum. These effects were associated with the improvement of peripheral insulin sensitivity and *β*-cell function and attributed to the polyphenolic profile—mainly composed by myricetin derivatives, as well as other flavonoids and tannins—identified in HESc [23]. More recently, we reported the HPLC-MS/MS phytochemical characterization of a polyphenol-rich extract (PESc) prepared from the aforementioned HESc, which allowed the identification of five main compounds as follows: gallic acid, myricetin, myricetin-3-*α*-arabinopyranoside, myricetin deoxyhexoside, and quercetin, with myricetin accounting for nearly 20% of PESc total polyphenol content [24]. Notwithstanding, PESc exhibited a strong antioxidant capacity against both biological and nonbiological oxidants, which enabled it to protect mice from an oxidative stress-induced diabetic state [24]. However, the molecular mechanisms responsible for the improvement of lipid metabolism promoted by *S. cumini* leaf remain poorly investigated.

Thus, taking into account our previous reports that hypertriglyceridemia in MSG-obese rats is associated with activation of the XBP-1s/PDI/MTP axis [8] and that HESc reverted their characteristic NAFLD and hypertriglyceridemia [23], in the present study, we sought to investigate the molecular mechanisms underlying the antihypertriglyceridemic activity of HESc in MSG-obese rats. The data presented herein endorse our hypothesis by presenting a novel feasible mechanism for the HESc antihypertriglyceridemic effect, which corroborates *S. cumini* leaf as a source of compounds for hypolipemiant purposes.

## 2. Materials and Methods

### 2.1. Plant Material

Leaves from *Syzygium cumini* (L.) Skeels, popularly known as jambolão in Brazil and java plum or black plum in English-spoken countries, were collected from specimens located at the Dom Delgado Campus (2°33′11.7^″^S 44°18′22.7^″^W) of the Federal University of Maranhão (UFMA; São Luís, Maranhão, Brazil). A voucher specimen was identified by Prof. Dr. Eduardo Bezerra Almeida Jr., a botanist at the Herbarium of Maranhão (MAR, Department of Biology, UFMA), and stored under the register number 4574. Furthermore, the species' name was confirmed in http://www.theplantlist.org on 08/15/2018.

### 2.2. Hydroethanolic Extract Preparation

**Figure 1:**
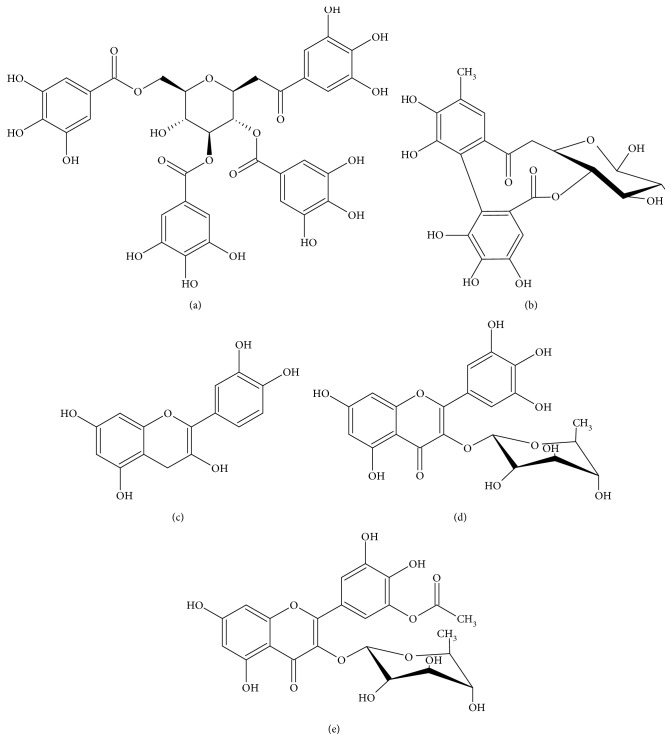
Main polyphenolic compounds identified in the hydroethanolic extract of *Syzygium cumini* leaves (HESc). (a) Tetragalloylglucose, (b) hexahydroxydiphenoyl-glucose, (c) quercetin, (d) myricetin deoxyhexoside, and (e) acylated myricetin deoxyhexoside.

After leaf collection, the hydroethanolic extract of *S. cumini* leaf (HESc) was obtained as previously described [23]. Upon lyophilization, HESc powder was stored at 4°C and freshly diluted in 0.9% NaCl at proper concentrations for oral administration to the animals. An aliquot of HESc was analyzed by HPLC-MS/MS to validate its authenticity. As shown in Supplementary Figure 1, HESc fingerprint corresponds to the same polyphenolic profile previously reported by us [23], whose main compounds are shown in Figure 1.

### 2.3. MSG Obesity Induction and Experimental Design

Newborn male Wistar rats were subcutaneously injected with the MSG solution (4.0 g/kg/day, Sigma-Aldrich, USA, Cat# G1626) or saline 1.25% (1.0 mL/kg/day), in accordance with our previous report [8]. From birth, all animals were kept under controlled conditions of temperature (23 ± 2°C) and light (12 h light/12 h dark) with filtered water and regular chow (CR-1 Nuvilab, Curitiba, Brazil) provided *ad libitum*. At 8 weeks of age, obesity development was assessed by calculating the Lee index (LI) (body weight (g)^1/3^/nasoanal length (cm) × 1000) [25]. MSG-obese rats and their appropriated lean controls were randomly divided into 4 groups and orally treated (gavage) as follows:

1. Lean: lean rats receiving 1.0 mL/kg/day saline 0.9% (*n* = 7)
2. Obese: MSG-obese rats receiving 1.0 mL/kg/day saline 0.9% (*n* = 7)
3. Obese+HESc 0.5: MSG-obese rats receiving 0.5 g/kg/day HESc (*n* = 7)
4. Obese+HESc 1.0: MSG-obese rats receiving 1.0 g/kg/day HESc (*n* = 7)

Body weight and food intake were measured twice a week throughout 8 weeks of treatment. At the end, the LI was again calculated to evaluate the effects of the treatment on body mass. Next, upon overnight fasting, animals were anesthetized (10 mg/kg xylazine+40 mg/kg ketamine, i.p.) for blood collection via abdominal aorta puncture and subsequent euthanasia by exsanguination. The liver and both retroperitoneal and periepididymal fat pads were collected, weighed, and appropriately stored for posterior assessments. All animal procedures were in accordance with the National Council for the Control of Animal Experimentation (CONCEA, Brazil) and approved by the Committee for Ethics and Welfare on Animal Use (CEUA) of UFMA under ruling number 23115.01983/2013-41.

### 2.4. Serum Biochemical Analysis and Assessment of Insulin Resistance

Glucose (GL), total cholesterol (TC), TG, FFA, aspartate aminotransferase (AST), and alanine aminotransferase (ALT) levels were assessed in serum samples using spectrophotometric commercial kits according to the manufacturers' instructions (Labtest, MG, Brazil, and Wako, VA, USA). Insulin resistance was inferred by calculating the TyG index (TyG = natural logarithm [fasting TG (mg/dL) × fasting GL (mg/dL)/2]) [26].

### 2.5. Liver Histological Analysis

Liver slides were obtained through 6 *μ*m thick transversal sections, stained with hematoxylin-eosin (HE), and assessed by 2 independent researchers in a double-blind way for the determination of the NAFLD activity score (NAS). This score is based on a semiquantitative analysis of the three definer criteria of NASH: steatosis (0-3), ballooning (0-3), and lobular inflammation (0-2). Total score is a value ranging from 0 to 8, which indicates a hepatic prognostic status. Scores > 6 indicate NASH; from 3 to 5, borderline; and from 0 to 2, it is not NASH [27].

### 2.6. Liver Lipid Profile

The hepatic lipid profile was assessed as previously described [28]. Briefly, a chloroform : methanol (2 : 1) solution was used to extract total fat from 500 mg liver samples, which were resuspended in a Triton-X100 : methanol (2 : 1) solution for the measurement of TG and TC levels as described in Section 2.4.

### 2.7. Chromatographic Analysis of Serum Lipoproteins

Serum lipoproteins were separated by fast protein liquid chromatography (FPLC) in a Superose 6 HR 10/30 column (Amersham Biosciences, Sweden) eluted with Tris buffer (pH 7.0; 10 mmol Tris, 150 mmol NaCl, 1 M EDTA, and 0.03% NaN_3_) at a rate of 0.5 mL/min, as previously described [29]. A total of 60 fractions were collected in a chronological order representing the density of each lipoprotein particle. Specifically, fractions 1-15 were labeled as VLDL, 16-30 were labeled as low-density lipoprotein (LDL), 31-45 were labeled as high-density lipoprotein (HDL), and 45-60 were labeled as other serum proteins. The TC and TG contents in each fraction were measured as described in Section 2.4. The total protein content was determined from absorbance at 280 nm.

### 2.8. Evaluation of Protein Expression by Western Blotting

Liver samples (*n* = 7) were homogenized by sonication with lysis buffer containing protease inhibitors (1 *μ*g/mL aprotinin, 1 *μ*g/mL leupeptin, and 10 mM PMSF). For each sample, 30 *μ*g of total protein was diluted with sample buffer and loaded into a SDS-PAGE gel for protein separation, which was transferred to nitrocellulose membranes. For the detection of the proteins of interest, membranes were incubated with primary antibodies: anti-KDEL (Enzo Life Sciences, USA, Cat# ADI-SPA-827), anti-XBP1 (Enzo Life Sciences, USA, Cat# ADI-905-739), anti-PDI (Enzo Life Sciences, USA, Cat# ADI-SPA-891), and anti-MTP (Sigma-Aldrich, USA, Cat# AV43618), followed by incubation with peroxidase-conjugated secondary antibodies for chemiluminescent detection (peroxidase-H_2_O_2_-luminol). *β*-Actin (Sigma-Aldrich, USA, Cat# A5441) was used as protein loading control.

### 2.9. Induction of Acute Hypertriglyceridemia with Triton WR1339

Eight-week-old male nonobese Wistar rats were randomized and administered with a single oral dose of saline 0.9% (0.1 mL/100 g) or either 0.5 g/kg or 1.0 g/kg HESc. After 1 hour, HESc-treated rats were intraperitoneally injected with 0.3 g/kg Triton WR1339 (Sigma-Aldrich, USA, Cat# T8761) and referred as the +HESc 0.5 g/kg and +HESc 1.0 g/kg groups (*n* = 7 per group). Saline-treated rats were injected with either equal Triton WR1339 dose (Triton WR1339 group, *n* = 7) or saline 0.9% (0.1 mL/100 g; control group, *n* = 7). Fasting blood samples were collected from the tail vein before (0 h) as well as 24, 48, and 72 h after the administration of Triton WR1339 for the determination of TG levels as described in Section 2.4 [30].

### 2.10. Statistical Analysis

Results were expressed as the mean ± standard error of the mean (SEM). The Shapiro-Wilk test was applied for normality assuring and groups compared by one-way analysis of variance (ANOVA) followed by the Newman-Keuls as posttest with Prism 7.0 (GraphPad, USA). Statistically significant differences were set at 5% with *p* < 0.05.

## 3. Results

### 3.1. HESc Reduces Adipose Tissue Accumulation in Obese Rats

**Figure 2:**
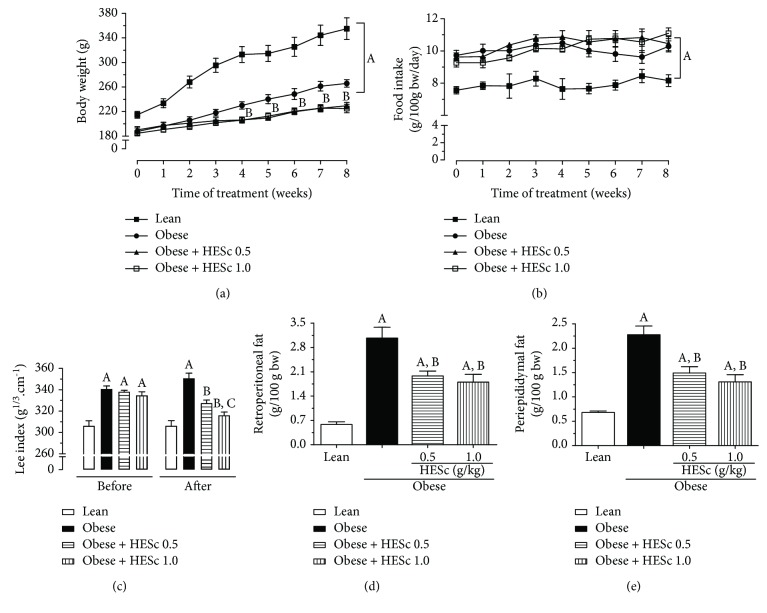
Administration of hydroethanolic extract of *Syzygium cumini* leaves (HESc) reduces fat accumulation in obese rats. (a) Evolution of body weight. (b) Daily food intake. (c) Lee index before and after 8 weeks of treatment. (d) Retroperitoneal fat. (e) Periepididymal fat. Lean: control group. Obese: obese group. Obese+HESc 0.5: obese animals treated with 0.5 g/kg of HESc. Obese+HESc 1.0: obese animals treated with 1.0 g/kg of HESc. Results are expressed as mean ± SEM (*n* = 7 per group). Letters indicate differences (*p* < 0.05; ANOVA; Newman-Keuls) as compared to ^a^lean, ^b^obese, and ^c^obese+HESc 0.5.

The lean group had higher mean body weight than the MSG-obese groups throughout the 8-week treatment period (Figure 2(a)), a peculiar feature of this animal model because of its shorter body length associated with deficient growth hormone (GH) secretion. However, MSG-obese rats presented a LI value 11% higher than the lean group (Figure 2(c)), accompanied by a 4-fold increase of retroperitoneal and periepididymal fat pads (Figures 2(d) and 2(e), respectively), denoting their obese condition. HESc treatment (0.5 and 1.0 g/kg/day) reduced the weight gain of obese rats by 15% regardless of the administered dose (Figure 2(a)), though no effect had been detected on food intake (Figure 2(b)). Besides, the LI was reduced by 7% and 10% in the obese+HESc 0.5 and obese+HESc 1.0 groups, respectively (Figure 2(c)). Weight loss was followed by a strong decrease of white adipose tissue accumulation, since retroperitoneal and periepididymal fat pads were, respectively, reduced by nearly 47% and 40% at both doses (Figures 2(d) and 2(e)).

### 3.2. HESc Improves Serum Glycolipid Profile in Obese Rats

**Figure 3:**
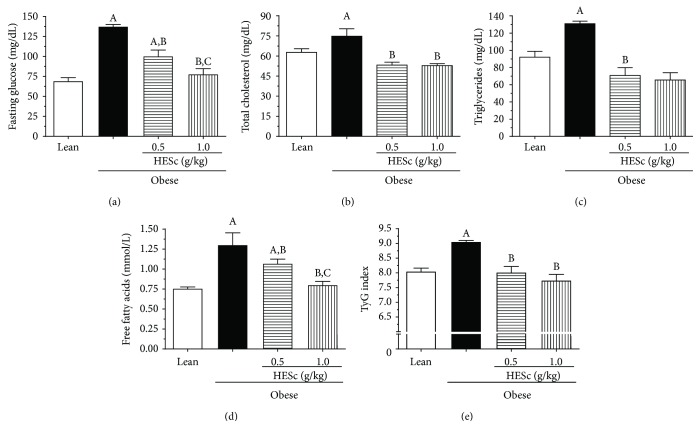
Administration of hydroethanolic extract of *Syzygium cumini* leaves (HESc) improves glucose homeostasis and serum lipid profile in obese rats. (a) Fasting glucose. (b) Total cholesterol. (c) Triglycerides. (d) Free fatty acids. (e) TyG index. Lean: control group. Obese: obese group. Obese+HESc 0.5: obese animals treated with 0.5 g/kg of HESc. Obese+HESc 1.0: obese animals treated with 1.0 g/kg of HESc. Results are expressed as mean ± SEM (*n* = 7 per group). Letters indicate differences (*p* < 0.05; ANOVA; Newman-Keuls) with respect to ^a^lean; ^b^obese, and ^c^obese+HESc 0.5.

At the end of treatment, obese rats presented serum fasting glucose levels 2-fold higher than the lean ones, which were dose-dependently reduced by 27% and 43% in the obese+HESc 0.5 and obese+HESc 1.0 groups, respectively (Figure 3(a)). The TC and TG levels of the HESc-treated groups were completely restored at both doses in relation to lean (Figures 3(b) and 3(c)). Circulating FFA levels on the HESc-treated groups were also reduced in a dose-dependent manner by 23% and 49% at doses of 0.5 and 1.0 g/kg, respectively, when compared to the MSG-obese group (Figure 3(d)). TyG index was used as a surrogate method for insulin sensitivity assessment. The data in Figure 3(e) indicates impaired insulin sensitivity on the MSG-obese group in relation to the lean group, which was precluded on HESc-treated obese animals.

### 3.3. HESc Reverts NAFLD and Improves Liver Function in Obese Rats

**Figure 4:**
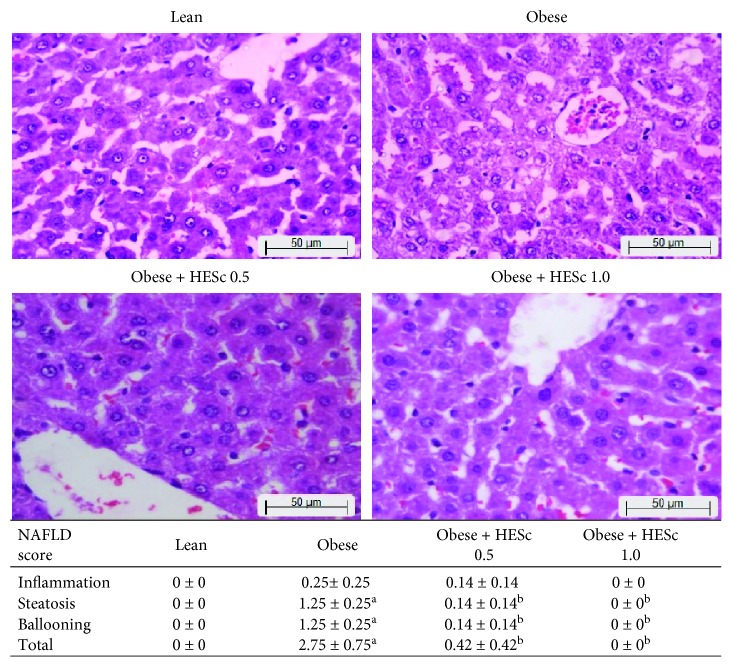
Administration of hydroethanolic extract of *Syzygium cumini* leaves (HESc) reverses nonalcoholic fatty liver disease (NAFLD) in obese rats. Analysis of liver histology with hematoxylin and eosin [27]. Lean: control group. Obese: obese group. Obese+HESc 0.5: obese animals treated with 0.5 g/kg of HESc. Obese+HESc 1.0: obese animals treated with 1.0 g/kg of HESc. Results are expressed as the mean ± SEM (*n* = 7 per group). Letters indicate differences (*p* < 0.05; ANOVA; Newman-Keuls) with respect to ^a^lean and ^b^obese.

**Figure 5:**
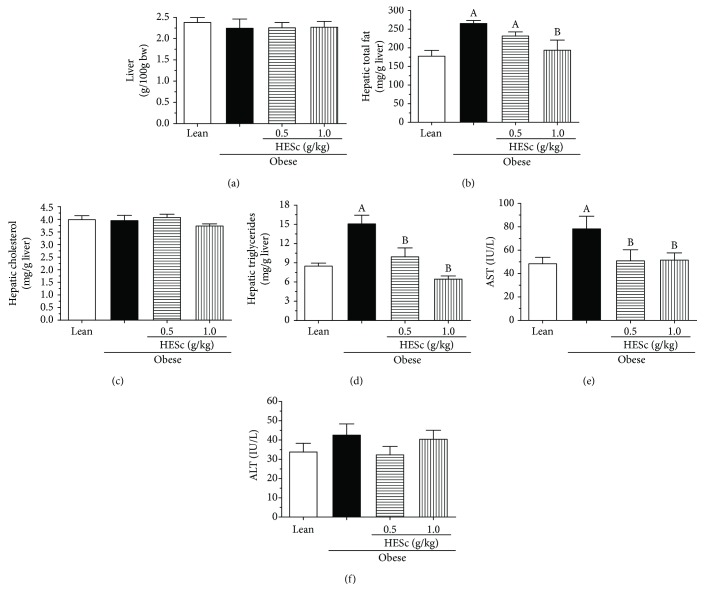
Administration of hydroethanolic extract of *Syzygium cumini* leaves (HESc) improves lipid profile and liver function of obese rats. (a) Liver weight. (b) Total liver fat. (c) Hepatic cholesterol. (d) Hepatic triglycerides. (e) Serum aspartate aminotransferase (AST). (f) Serum alanine aminotransferase (ALT). Lean: control group. Obese: obese group. Obese+HESc 0.5: obese animals treated with 0.5 g/kg of HESc. Obese+HESc 1.0: obese animals treated with 1.0 g/kg of HESc. Results are expressed as the mean ± SEM (*n* = 7 per group). Letters indicate differences (*p* < 0.05; ANOVA; Newman-Keuls) with respect to ^a^lean and ^b^obese.

As shown in Figure 4, the NAS score for obese animals supported the presence of hepatic steatosis and cell ballooning and suggested low-grade inflammation, as compared to lean rats, putting those animals in an intermediate way toward nonalcoholic steatohepatitis (NASH). However, the administration of HESc reverted these features in both groups. Noteworthy, obese+HESc 1.0 animals did not score in any assessed parameters. Histological analysis was corroborated by data from the liver lipid profile. Even though no difference had been seen in the livers' relative weights (Figure 5(a)), the total fat content was increased by nearly 50% in the obese group, as compared to lean. Again, HESc reduced such fat accumulation by 13% in obese+HESc 0.5 and 27% in obese+HESc 1.0, as compared to the obese group (Figure 5(b)). Alike, the TG levels were increased by 76% in obese animals as compared to the lean group but reduced by 44% and 57% in the obese+HESc 0.5 and obese+HESc 1.0 groups, respectively (Figure 5(d)). No difference was observed in the liver cholesterol content among the groups (Figure 5(c)). To evaluate to which extent this fat accumulation impaired liver function, the activities of AST and ALT were assessed. As Figure 5(e) shows, there was a 60% increase in AST activity on obese rats compared to the lean group, whereas HESc reduced its activity by nearly 35% at both doses. On the other hand, there was no change in ALT activity among the groups (Figure 5(f)).

### 3.4. HESc Reduces the TG Content in VLDL Particles from Obese Rats

**Figure 6:**
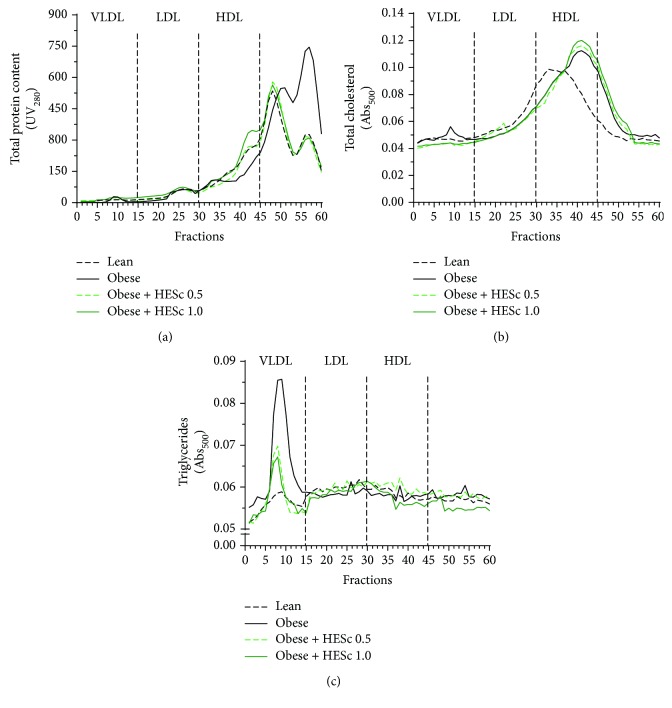
Administration of hydroethanolic extract of *Syzygium cumini* leaves (HESc) reduces the triglyceride content in VLDL particles of obese rats. (a) Protein content of high-density lipoprotein (HDL), low-density lipoprotein (LDL), and very low-density lipoprotein (VLDL). (b) Cholesterol content of lipoproteins. (c) Triglyceride content of lipoproteins. Lean: control group. Obese: obese group. Obese+HESc 0.5: obese animals treated with 0.5 g/kg of HESc. Obese+HESc 1.0: obese animals treated with 1.0 g/kg of HESc. Results are expressed as the mean absorbance of fast protein liquid chromatography (FPLC) (*n* = 7 per group).

FPLC analysis of serum samples showed that the nonlipoprotein particle-bound protein content (fractions 45-60) of obese animals was 48% higher than in lean animals, which was fully restored in the animals treated with HESc (Figure 6(a)). In fractions corresponding to HDL particles (fractions 30-45), the protein content was 30% lower in obese animals as compared with lean animals, whereas treatment with HESc at 0.5 and 1.0 g/kg increased these levels by 30% and 72%, respectively, in comparison with the former (Figure 6(a)). Likewise, the cholesterol content in HDL fractions from the obese group exhibited 36% higher levels than the lean group. HESc treatment did not change the cholesterol content in HDL particles but restored it in VLDL particles (fractions 1-15; Figure 6(b)). Noteworthy, VLDL particles from obese rats contained 3-fold more TG than the lean group. HESc dropped down these levels by nearly 50% in both treated groups (Figure 6(c)).

### 3.5. HESc Reduces ER Stress in the Liver of Obese Rats

**Figure 7:**
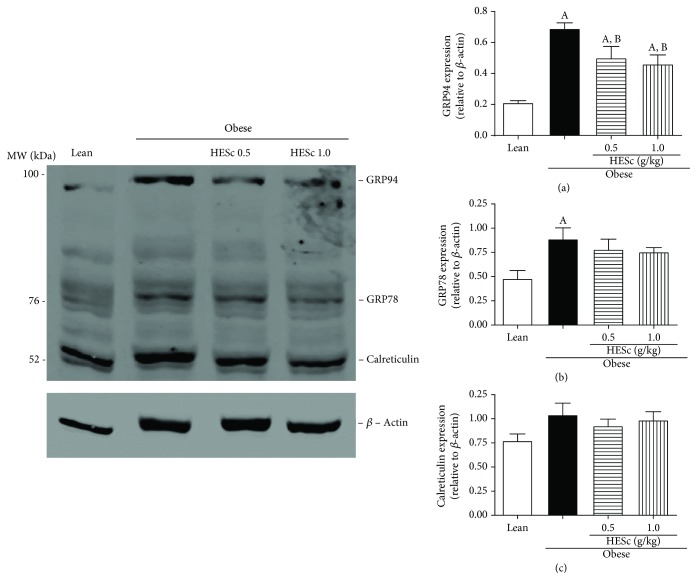
Administration of hydroethanolic extract of *Syzygium cumini* leaves (HESc) attenuates endoplasmic reticulum stress in the livers of obese rats. Protein expression was determined by western blotting. (a) Glucose response protein 94 (GRP94). (b) Glucose response protein 78 (GRP78). (c) Calreticulin. Lean: control group. Obese: obese group. Obese+HESc 0.5: obese animals treated with 0.5 g/kg of HESc. Obese+HESc 1.0: obese animals treated with 1.0 g/kg of HESc. Densitometry results are expressed as the mean ± SEM (*n* = 4 per group). Letters indicate differences (*p* < 0.05; ANOVA; Newman-Keuls) with respect to ^a^lean and ^b^obese.

Measurement of the translational levels of KDEL chaperones, which are involved in hepatic UPR, exhibited a 3-fold increase for GRP94 on obese rats as compared to lean. HESc treatment reduced this protein expression by 27% and 33% at doses of 0.5 and 1.0 g/kg, respectively. On the other hand, GRP78 expression was increased by 83% in the obese group, but only marginally reduced in HESc-treated animals, since the difference did not reach statistically significant values. Likewise, there was no difference in calreticulin expression among groups (Figure 7).

### 3.6. HESc Inhibits the XBP-1s/PDI/MTP Axis

**Figure 8:**
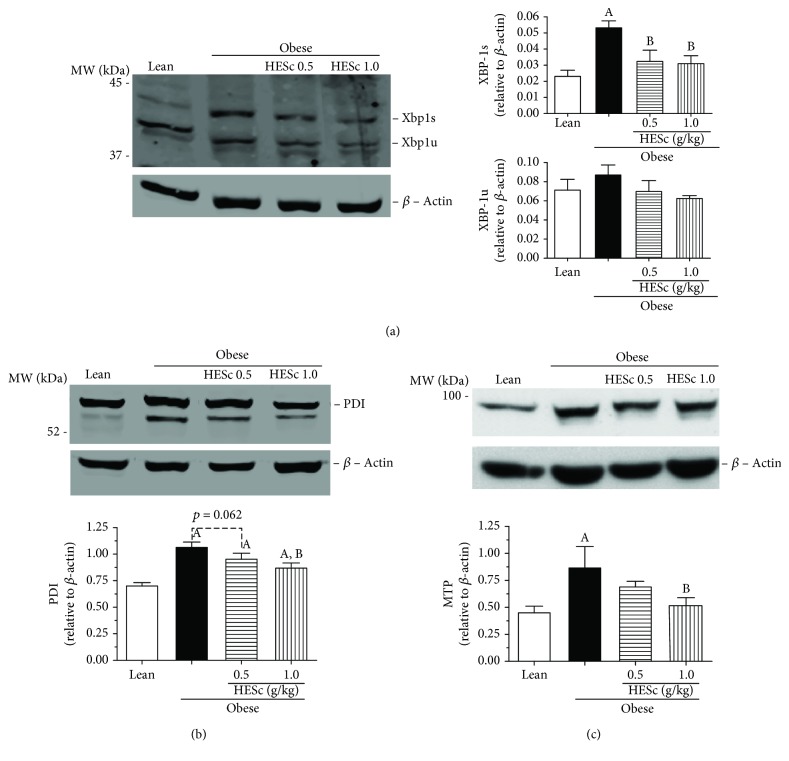
Administration of hydroethanolic extract of *Syzygium cumini* leaves (HESc) inhibits the XBP-1s/PDI/MTP pathway in the livers of obese rats. Protein expression was determined by western blotting. (a) X-box binding protein 1 (XBP-1). (b) Protein disulfide isomerase (PDI). (c) Microsomal triglyceride-transfer protein (MTP). Lean: control group. Obese 0: obese group. Obese+HESc 0.5: obese animals treated with 0.5 g/kg of HESc. Obese+HESc 1.0: obese animals treated with 1.0 g/kg of HESc. Densitometry results are expressed as the mean ± SEM (*n* = 4 per group). Letters indicate differences (*p* < 0.05; ANOVA; Newman-Keuls) with respect to ^a^lean and ^b^obese.

**Figure 9:**
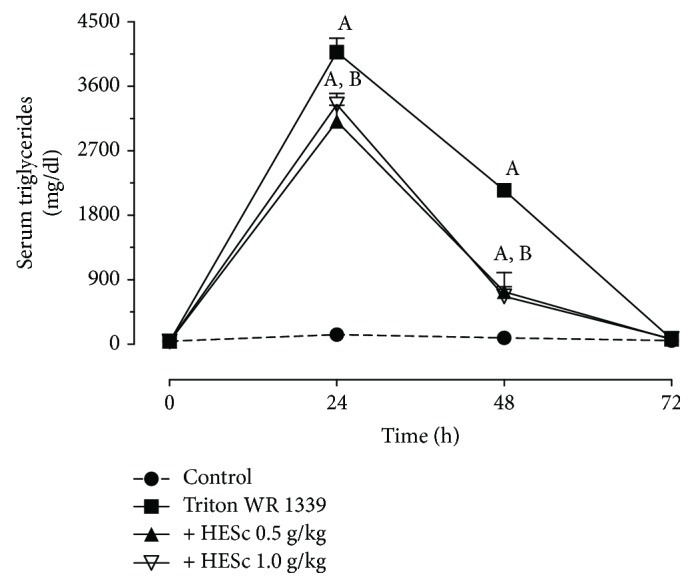
Administration of hydroethanolic extract of *Syzygium cumini* leaves (HESc) reduces the export of liver triglycerides in rats with Triton WR1339-induced acute hypertriglyceridemia. Acute hypertriglyceridemia was induced by an intraperitoneal injection of Triton WR1339 (0.3 g/kg) 1 hour after a single dose of HESc was administered, and the rats were monitored for 72 h. Control: normotriglyceridemic group. Triton WR1339: hypertriglyceridemic group. +HESc 0.5: hypertriglyceridemic group treated with 0.5 g/kg of HESc. +HESc 1.0: hypertriglyceridemic group treated with 1.0 g/kg of HESc. Results are expressed as the mean ± SEM (*n* = 7 per group). Letters indicate differences (*p* < 0.05; ANOVA; Newman-Keuls) with respect to ^a^lean and ^b^obese.

Assessment of XBP-1 protein expression for both spliced (XBP-1s) and unspliced (XBP-1u) forms revealed a 2.5-fold higher splicing rates in MSG-obese rats than in the lean group. This increase was reduced to values similar to those of the lean group upon treatment with both doses of HESc (Figure 8(a)). PDI expression was increased by 52% on obese animals but brought back to intermediate levels in the obese+HESc 1.0 group, with a reduction of 21% (Figure 8(b)). Similarly, MTP expression was 2-fold higher on the obese group and reduced to lean-like levels in animals from the obese+HESc 1.0 group (Figure 8(c)). In order to verify the effect of HESc on MTP function, this enzyme's activity was assessed in nonobese rats acutely injected with Triton WR1339, which is a well-known model of MTP-mediated hypertriglyceridemia [30]. Oral administration of HESc, at the same abovementioned doses, 1-hour prior induction reduced serum TG accumulation within 24 h, as well as hastened its clearance in the following 48 h (Figure 9), which is in line with a lower rate of VLDL particles assembly and secretion from the liver.

## 4. Discussion

This study strengthens *S. cumini* pharmacological potentialities since it corroborates our previous report that HESc restores serum TG levels in hypertriglyceridemic MSG-obese rats [23]. The data presented herein expand these findings by showing that oral administration of HESc to MSG-obese rats for 8 weeks detains weight gain, improves fatty liver disease, and reverts hypertriglyceridemia, besides other metabolic outcomes typically found in this MetS rodent model. Specifically, this study shows that HESc inhibited both expression and activity of hepatic MTP by downregulation of the XBP-1s/PDI/MTP axis, reducing the incorporation of TG into VLDL particles and consequently lowering the circulating TG levels.

Neonatal administration of MSG damages hypothalamic nuclei, e.g., arcuate nucleus, leading to impaired GH secretion; therefore, adult animals are shorter and lighter than age-matched controls but present higher fat mass [31]. Furthermore, these animals also exhibit autonomic unbalance characterized by enhanced vagus nerve tonus, which imposes increased insulin secretion and consequent development of peripheral insulin resistance and elevation of fat stores [32]. In this study, treatment with HESc reduced body weight gain in obese rats but did not affect their food intake. Although an extract from *S. cumini* leaf had been shown to decrease food intake on nonobese rats [33], the lower weight gain displayed by our treated obese rats is most likely related to the lipolytic action of HESc, as we previously described [23]. HESc is particularly rich in tetragalloylglucose (Figure 1(a)), a gallotannin whose lipolytic effects have been attributed to the modulation of proliferative peroxisome-activated receptor gamma (PPAR*γ*) [34], a mechanism shared by the compound vitalbosine A isolated from *S. cumini* seed [35].

Besides antiobesity effects, HESc also improved the serum lipid profile of obese rats. Particularly, it reverted the remarkable hypertriglyceridemia peculiarly displayed by MSG-obese rats [8], an effect further extensive to serum TC and FFA levels. In accordance, the TG content into VLDL particles from HESc-treated obese rats was halved in comparison to nontreated obese animals. HESc also reduced the excess ectopic liver fat in obese rats, an effect associated to TG but not to cholesterol content. This lipid-lowering effect of HESc seems to be responsible for the complete restoration of the hepatic histopathological pattern of obese rats, whose NAS score was brought back to values very similar to lean healthy animals. These effects might be related to improved hepatic insulin sensitivity promoted by HESc, which is supported by the reduced TyG index value found in treated obese animals. Importantly, the TyG index has been proposed as a biomarker of NAFLD initiation and progression even in asymptomatic subjects [36]. Studies carried out with *S. cumini* seed extract in HepG2 cells [37] and livers from streptozotocin-induced diabetic rats [38] have attributed its hypolipidemic effect to increased PPAR*γ* activity and expression. Myricetin (Figures 1(d) and (e)), the most prevalent flavonoid in HESc [23], has been shown to improve insulin sensitivity [39] and promote hepatic lipid oxidation by increasing PPAR*α* expression in the liver [40].

In addition to the extensive knowledge on *S. cumini* effects on peripheral insulin sensitivity, particularly on the PPAR*α*/*γ* pathways, we hypothesize that HESc polyphenols might also interfere with the ER stress-sensing IRE1*α*/XBP-1s pathway, which has also been proposed as an important mechanism underlying the development of NAFLD and hypertriglyceridemia, as demonstrated in hepatocyte-specific IRE1*α*-null mice [11] and MSG-obese rats [8].

In the past decade, hepatic ER stress has been proposed as a main contributing factor for NAFLD onset and progression, as well as MetS-associated dyslipidemias [5, 41]. Initial UPR is characterized by elevated gene/protein expression of KDEL chaperones, namely, glucose-regulated protein 94 (GRP94), GRP78, and calreticulin, to mitigate protein misfolding and reestablish ER homeostasis within a negative feedback loop regulated by both the IRE1*α* and ATF6 pathways [41, 42]. Our obese rats showed a clear increase of hepatic GRP94 and GRP78 protein expressions, denoting active UPR, which was partially attenuated in HESc-treated obese rats. Many actions of HESc, such as improvement of insulin sensitivity and lower FFA circulating levels, might also be involved in this effect since it has been shown that increased serum FFA levels might induce hepatic ER stress [43], meanwhile polyphenols such as myricetin derivatives are able to attenuate it [44].

Studies have demonstrated that the IRE1*α*/XBP-1s pathway is the most conserved arm of UPR [45], which is importantly involved in the control of glucose homeostasis and lipid metabolism [9, 10]. Regardless of a recent discussion about its lipogenic [10] or antilipogenic role [46] in the liver, it is well established that XBP-1s acts as a hypertriglyceridemic factor [7, 46]. XBP-1s activates the expression of MTP and its active subunit PDI, which favors the incorporation of TG into nascent VLDL particles [11]. Noteworthy, the function of XBP-1 on hepatic lipogenesis is unrelated to its function in the UPR but nevertheless requires its splicing by IRE1*α* [7]. Here, HESc downregulated this pathway, since obese-treated animals presented lower splicing of XBP-1s along with lesser expression of both PDI and MTP, as compared to obese nontreated rats. The inhibitory effect of HESc on the XBP-1/PDI/MTP axis was further supported by its acute action on Triton WR1339-induced hypertriglyceridemic rats, which not only reduced total TG accumulation within 24 h but also hastened its clearance. Alike, a recent study has shown the inhibitory effect of polyphenols from *Punica granatum* flower on the IRE1*α*/XBP1s pathway [47]. To the best of our knowledge, this is the first study to describe a feasible molecular mechanism underlying the antihypertriglyceridemic properties of *S. cumini*.

There is consistent evidence about the effects of polyphenol-rich extracts or polyphenols per se on the ER stress pathways. Preincubation of polyphenol-rich extract of *Vitis rotundifolia* has been shown to inhibit thapsigargin-induced ER stress in human retinal endothelial cells [48]. Administration of polyphenol-rich extracts from pomegranate and green tea, for 20 weeks, to high-fat diet obese mice attenuated UPR activation in the skeletal muscle by reducing the gene expression of GRP78 and XBP1 splicing [49], an action also described for kaempferol in ischemic cardiomyocytes [50] and for apigenin in neuronal cells with ER stress induced by thapsigargin or brefeldin A [51]. In addition, myricetin and its derivatives have been shown to decrease protein expression of ER stress markers, such as GRP78 and IRE1*α* in the colon of mice with colitis-associated cancer [44]. Notwithstanding, since oxidative stress is enrolled as UPR inducer [52], polyphenols contained in HESc, particularly myricetin derivatives, would also have attenuated ER stress by reestablishing cellular redox balance in the liver of our treated MSG-obese rats, an obesity model whose oxidative stress has already been characterized [53, 54]. Thus, future studies must specifically address this issue as well as assess the effects of isolated compounds in order to pursue additional mechanisms and identify the molecules probably responsible for them.

## 5. Conclusions

In conclusion, the data presented herein reinforce the prominent metabolic properties of *S. cumini* leaf. The recovery of normal serum TG levels on HESc-treated obese rats discloses a novel feasible mechanism of action for the hypolipemiant effect traditionally ascribed to this plant species via inhibition of the hepatic XBP-1s/PDI/MTP axis. Hepatic MTP inhibitors have been considered important agents to treat familial dyslipidemia, as that seen in abetalipoproteinemia, but their clinical utility has been restricted by the increased risk of hepatic steatosis [55, 56]. In addition to its antihypertriglyceridemic effect, HESc also restored the hepatic fat accumulation of obese rats. This secondary, but not less important, property supports the possibility of synergism among the mechanism shown herein and other properties previously described for HESc, such as huge antioxidant capacity and improvement of peripheral insulin sensitivity [23, 24]. Finally, this dual action further corroborates the multitarget potentialities of the polyphenols contained into HESc as a potential phytocomplex against MetS-derived metabolic disturbances.

## Data Availability

The data used to support the findings of this study are available from the corresponding author upon request.

## Supplementary Materials

Supplementary Materials Figure S1: HPLC fingerprint of the hydroethanolic extract of *S. cumini* leaf (HESc). Representative HPLC chromatogram with UV detection at 254 nm of HESc, which was performed in triplicate for the validation of its authenticity in accordance with the analysis previously reported (Sanches et al., [23]).
