# Supplementary material for: Syzygium cumini Leaf Extract Reverts Hypertriglyceridemia via Downregulation of the Hepatic XBP-1s/PDI/MTP Axis in Monosodium L-Glutamate-Induced Obese Rats
Source: Oxid Med Cell Longev. 2019 Mar 21;2019:9417498. doi: 10.1155/2019/9417498 (PMC6446099; doi:10.1155/2019/9417498)
Supplement: Supplementary Materials — Figure S1: HPLC fingerprint of the hydroethanolic extract of S. cumini leaf (HESc). Representative HPLC chromatogram with UV detection at 254 nm of HESc, which was performed in triplicate for the validation of its authenticity in accordance with the analysis previously reported (Sanches et al., [23]). [file 9417498.f1.pdf]

SUPPLEMENTARY MATERIAL

Research Article

Syzygium cumini leaf extract reverts hypertriglyceridemia via downregulation of the hepatic XBP-1s/PDI/MTP axis in monosodium L-glutamate-induced obese rats

Authorship

Lucas Martins França[1], Caio Fernando Ferreira Coêlho[1], Larissa Nara Costa Freitas[1], Ivana Letícia Santos Souza[1], Vinicyus Teles Chagas[1], Victor Debbas[2], Thais Martins de Lima[3], Heraldo Possolo de Souza[3], Francisco Rafael Martins Laurindo[2], Antonio Marcus de Andrade Paes[1*]

[1]aboratory of Experimental Physiology (LeFisio), Department of Physiological Sciences, Federal University of Maranhão, Av. dos Portugueses, 1966 – Cidade Universitária Dom Delgado, São Luís - MA, 65080-805, Brazil.

[2]Laboratory of Vascular Biology (LBV), Heart Institute, School of Medicine, University of São Paulo, Av. Dr. Enéas de Carvalho Aguiar, 44 – Cerqueira César, São Paulo - SP, 05403-900, Brazil.

[3]Laboratory of Medical Investigation (LIM-51), Emergency Medicine Department, School of Medicine, University of São Paulo, Av. Dr. Arnaldo, 455 - Cerqueira César, São Paulo - SP, 01246-903, Brazil

Validation of HESc polyphenolic profile by HPLC.

To validate the identity of HESc polyphenolic profile, in accordance to our previous report (Sanches et al., 2016), a chromatograph (LC-20A Prominence, Shimadzu) equipped with autoinjector and detector SPD-20A/UV-Vis was used for polyphenols separation. HESc was dissolved in methanol (500 µg/mL) and filtered through 0.45 mm mesh (Millipore) for an injection volume of 20 µL into an analytical column Luna C-18 (250 x 4.6 mm - 5 µm, Phenomenex). The mobile phase consisted of 0.1% formic acid acidified water (A) and acetonitrile (B) according to the following elution gradient: 0-1 min, 5% B; 1-30 min, 5%-30% B; 30-60 min, 30%-70% B; and flow rate of 1 mL/min at 40 °C oven to allow the fingerprint detection at 254 nm. All solvents were purchased from Merck Millipore Co. (Germany) and degassed using ultrasonic bath before the runs to be performed in triplicate. Supplementary Figure 1 shows a representative fingerprint of HESc HPLC analysis, which corresponded to the same polyphenolic profile previously reported by us (Sanches et al., 2016).

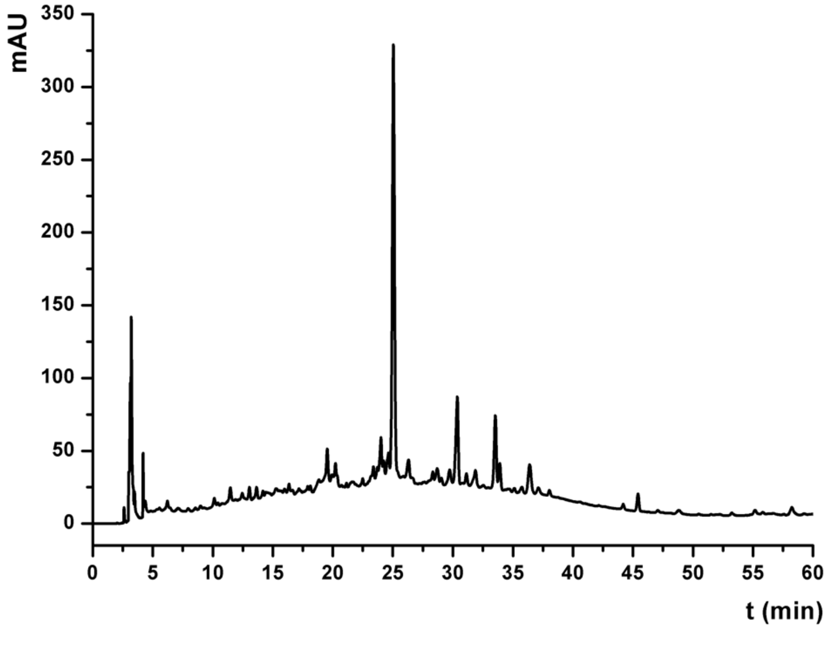

Supplementary Figure 1: HPLC fingerprint of the hydroethanolic extract of *S. cumini* leaf (HESc). Representative HPLC chromatogram with UV detection at 254 nm of HESc, which was performed in triplicate for validation of its authenticity in accordance with analysis previously reported (Sanches et al., 2016).

Reference

Sanches, J.R., Franca, L.M., Chagas, V.T., Gaspar, R.S., Dos Santos, K.A., Goncalves, L.M., Sloboda, D.M., Holloway, A.C., Dutra, R.P., Carneiro, E.M., Cappelli, A.P., Paes, A.M., 2016. Polyphenol-Rich Extract of Syzygium cumini Leaf Dually Improves Peripheral Insulin Sensitivity and Pancreatic Islet Function in Monosodium L-Glutamate-Induced Obese Rats. Frontiers in pharmacology 7, 48.
